# Panniculite au cours d'un traitement d'une dermatomyosite par du méthotrexate

**DOI:** 10.11604/pamj.2016.23.149.8950

**Published:** 2016-03-31

**Authors:** Nabil Belfeki, Monia Smiti Khanfir, Imed Ben Ghorbel, Fatma Said, Mohamed Habib Houman

**Affiliations:** 1 Service de Médecine Interne, Centre Hospitalo-Universitaire la Rabta, Tunis, Tunisie

**Keywords:** Dermatomyosite, panniculite, méthotrexate, Dermatomyosite, panniculite, méthotrexate

## Abstract

La panniculite est une manifestation rare au cours des dermatomyosites (DM). L'apparition d'une panniculite au cours d'un traitement par du méthotrexate (MTX) est exceptionnelle et n'a été décrite que dans 3 cas. Nous rapportons l'observation d'une patiente âgée de 50 ans atteinte d'une DM diagnostiquée en 1997 et traitée par une corticothérapie avec une évolution favorable aux plans clinique et biologique. A l'occasion d'une rechute 2 ans plus tard, la corticothérapie a été majorée et du méthotrexate à une dose hebdomadaire de 7,5 mg a été rajouté. L’évolution était rapidement favorable. Dix-huit mois plus tard, la patiente présentait de multiples nodules sous cutanés siégeant aux 4 membres et aux fesses, dont l'examen anatomopthologique concluait à une panniculite. Il n'existait aucun signe d’évolutivité de la DM. La dose de prédnisone a été augmentée à 0,5 mg/kg/j toujours en association au MTX mais sans aucune amélioration. Le MTX a été arrêté et les lésions cutanées ont complètement disparu en 2 mois sans aucune récidive avec un recul actuel de 42 mois. Notre observation est particulière par la survenue d'une panniculite chez une patiente ayant une DM traitée par du MTX et illustre la difficulté diagnostique. Cette entité doit être connue malgré son caractère exceptionnel puisque l'arrêt du MTX induit en général la disparition des nodules sous cutanés.

## Abstract (en)

Panniculitis is a rare cutaneous manifestation of dermatomyositis (DM). The appearance of panniculitis during treatment with methotrexate (MTX) is exceptional and has only been described in 3 cases. We report a case of a 50-year-old woman suffering from DM since 1997 who was treated with corticosteroids showing favorable clinical and biological evolution. When a relapse occurred 2 years later, she was treated with higher-dose of corticosteroids in combination with a 7,5 mg weekly dose of methotrexate. The evolution was rapidly favorable. Eighteen months later, the patient had multiple subcutaneous nodules on limbs and buttocks. Anatomopathological examination showed panniculitis. There was no evidence supporting progression in DM. Prednisone dose was increased to 0.5 mg/kg/day, always in combination with MTX, without any clear signs of improvement. MTX treatment was stopped and the cutaneous lesions completely disappeared in 2 months without any relapse. This objective response lasted for 42 months. Our observation is particular given the occurrence of panniculitis in a patient undergoing treatment for dermatomyositis with methotrexate and illustrates the difficulties in the diagnosis. This entity must be known despite its exceptional nature since cutting off MTX treatment generally induces the disappearance of subcutaneous nodules.

## Introduction

La panniculite est rarement décrite parmi les manifestations cutanées de la dermatomyosite (DM). Elle survient le plus souvent au cours de la phase initiale de la maladie, témoignant de la gravité de la DM. La survenue d'une panniculite au cours d'un traitement par du méthotrexate (MTX) n'a été rapportée que dans trois cas [1–3]. Nous rapportons une observation d'une panniculite survenant au cours de l’évolution d'une DM traitée par du MTX et en phase de rémission.

## Patient et observation

**Figure 1:**
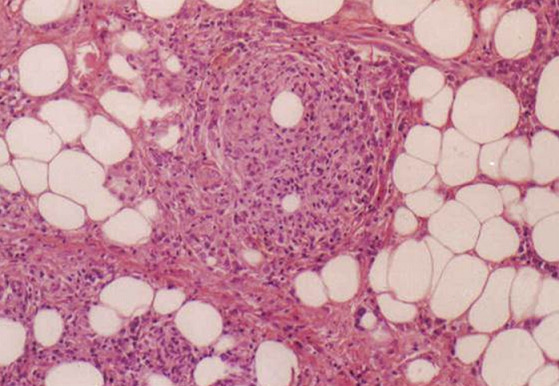
Infiltrat inflammatoire polymorphe composé de lymphocytes, de plasmocytes, de cellules épithélioïdes et de cellules géantes macrophagiques, groupés en follicules au niveau du tissu adipeux hypodermique

Patiente âgée de 50 ans a présenté en juillet 1997 un érythrœdème périorbitaire associé à une altération de l’état général. Elle se plaignait d'arthralgies de type inflammatoire intéressant les grosses articulations et de troubles de la déglutition. L'examen notait un œdème violacé périorbitaire bilatéral et des lésions érythémateuses squameuses au niveau du front, du décolleté, des épaules et des avant-bras. Un déficit musculaire proximal au niveau des ceintures scapulaires et pelviennes coté à 2 au testing musculaire et des myalgies spontanées et à la pression des masses musculaires ont été objectivées. Il existait un syndrome inflammatoire biologique avec une vitesse de sédimentation à 68 mm à la première heure, une fibrinémie à 4,85 mg/l et une hyperalpha2 globulinémie à 10,2 g/l. Les enzymes musculaires étaient augmentées: la créatine phosphokinase (CPK) à 1085 UI/L (5 fois la normale), la lacticodéshydrogénase (LDH) à 1036 UI/L, la transaminase glutamo-oxaloacétique (SGOT) à 94 UI/L et la transaminase glutamo-pyruvique (SGPT) à 39 UI/L. Le dosage de l'aldolase n'a pas été pratiqué. Le bilan immunologique révélait des anticorps antinucléaires à 1/800 de type moucheté. Les anticorps anti DNA natif et anti J01 étaient négatifs. L’électromyogramme mettait en évidence une activité de repos à type de fibrillations et de salves pseudomyotoniques témoignant d'une atteinte myogéne. L'examen anatomopathologique d'une biopsie musculaire révélait un tissu musculaire strié constitué de fibres d’épaisseur inégale avec par place des noyaux en chaînettes. Ces fibres étaient altérées avec une clarification floconneuse et une vacuolisation cytoplasmique. Le tissu interstitiel était dépourvu de fibrose ou d'infiltrat inflammatoire. Le diagnostic de dermatomyosite a été retenu. La radiographie du thorax et l’échographie cardiaque étaient sans anomalie. Un bilan exhaustif à la recherche d'une néoplasie associée comportant une échographie abdomino-pelvienne, une échographie cervicale, un scanner thoraco-abdomino-pelvien, une mammographie, un frottis cervico-vaginal, une fibroscopie digestive et une nasofibroscopie avec des biopsies du cavum était sans anomalie. Les marqueurs tumoraux (ACE, alpha foetoproteine, CA 125 et CA 19-9) étaient négatifs. La patiente a été traitée par de la prédnisone à la dose de 1 mg/kg/j. L’évolution a été favorable aux plans clinique et biologique avec une normalisation des enzymes musculaires au bout de 2 mois de traitement. Les doses de prédnisone ont été diminuées progressivement. Deux ans plus tard, la patiente a présenté une rechute de sa dermatomyosite alors qu'elle était traitée par une dose journalière de 10 mg de prédnisone. L'examen trouvait un érythrœdème en lunette associé à un érythème en bande au niveau des deux mains et des papules de Gottron. Un déficit musculaire des ceintures, des myalgies et une polyarthrite étaient objectivés. Les enzymes musculaires étaient augmentées (CPK = 122 UI/L, LDH = 728 UI/L). La corticothérapie a été augmentée à 1mg/kg/j associée à du méthotrexate à la dose hebdomadaire de 7,5 mg. L’évolution a été rapidement favorable. Dix huit mois plus tard, alors qu'elle était traitée par 5 mg de prédnisone par jour et 7,5 mg de méthotrexate par semaine, la patiente s'est plainte de l'apparition de multiples nodules sous cutanés, siégeant aux quatre membres et aux fesses. Ces nodules étaient douloureux, érythémateux, de taille variable et n’évoluant pas vers les différents stades de la biligénie. Il existait une lipoatrophie nette au niveau de chaque élément. L'examen physique notait un discret déficit de la ceinture scapulaire. Il n'existait pas de syndrome inflammatoire biologique ni d’élévation des enzymes musculaires. L'examen anatomopathologique de la biopsie d'un nodule sous cutané révélait l'existence d'un infiltrat inflammatoire polymorphe composé de lymphocytes, de plasmocytes, de cellules épithélioïdes et de cellules géantes macrophagiques, parfois groupés en follicules siégeant au niveau du tissu adipeux hypodermique et des septa fibreux (Figure 1). Les vaisseaux sanguins possédaient une paroi épaissie avec une turgescence des cellules endothéliales. Le diagnostic d'une panniculite associée à une DM a été retenu. Les doses de prédnisone ont été augmentées à 0,5 mg/kg/j toujours en association avec le MTX. L’évolution était marquée par la persistance des lésions cutanées et l'absence de manifestations cliniques ou biologiques d’évolutivité de la DM. Le rôle du MTX a été suspecté et ce traitement a été arrêté. L’évolution s'est faite vers la disparition des lésions cutanées en 2 mois sans récidive avec un recul actuel de 42 mois.

## Discussion

Le diagnostic de dermatomyosite a été porté chez notre patiente devant l'association des 5 critères diagnostiques de Bohan et Peter [4]. La panniculite a été diagnostiquée environ 4 ans après l'apparition des premières manifestations en rapport avec la DM. L'association d'une panniculite symptomatique et d'une DM est très rare. En effet, depuis sa première description en 1924 par weber et Gray [5], cette association n'a été rapportée à notre connaissance que dans 18 cas [6–21]. Dans la majorité des cas, la panniculite était de survenue concomitante avec les premières manifestations de la DM ou au cours des poussées de la maladie contrairement à notre observation puisque la panniculite était découverte en phase de rémission complète clinique et biologique de la DM. Il s'agissait de femmes âgées entre 50 et 65 ans dans la plupart des cas rapportés, à part quelques observations pédiatriques [9, 18, 20]. L’évolution était le plus souvent favorable après traitement par des corticoïdes associés dans certains cas à l'azathioprine ou la ciclosporine [16, 17]. Aucun de ces patients n’était traité par du MTX. Le MTX était incriminé dans la survenue ou l'aggravation de nodules rhumatoïdes dans 8 à 10% des cas de polyarthrite rhumatoïde [2]. Par contre, l'apparition d'une panniculite au cours du traitement par du MTX est une situation très rare qui n'a été décrite à notre connaissance que dans 3 cas seulement [1–3]. Il s'agissait d'une panniculite au cours du traitement par du MTX d'un rhumatisme psoriasique [3], d'une dermatomyosite [2] et d'un syndrome de Sharp [1]. Les mécanismes physiopathologiques de la panniculite iatrogène au MTX restent indéterminés. Dans le premier cas de Berris [3] la survenue des nodules mettait en cause le diagnostique de rhumatisme psoriasique en faveur d'une polyarthrite rhumatoïde où il était « habituel » d'observer une apparition ou une aggravation des nodules rhumatoïdes chez les patients traités par du MTX. Cependant, les lésions cutanées du psoriasis étaient évidentes, le facteur rhumatoïde était négatif et l'examen anatomopathologique d'un nodule cutané objectivait une panniculite septale. En 1999, Jang [2] rapportait une panniculite chez une patiente de 50 ans atteinte d'une dermatomyosite et traitée par du MTX. Les nodules cutanés étaient apparus 14 mois après le début du traitement par le MTX, initialement situés au niveau du décolleté, puis rapidement une extension vers le cou, les creux axillaires et les membres supérieurs a été observée. L'examen anatomopathologique objectivait une panniculite septale. Les nodules ont totalement disparu après l'arrêt du MTX et le traitement par de l'hydroxychloroquine. La panniculite observée chez notre patiente peut être en rapport avec la DM ou secondaire au traitement par le MTX. Cette deuxième éventualité semble la plus probable. En effet, les lésions cutanées de la panniculite sont apparues après l'introduction du MTX et à un moment où la DM était quiescente. D'autre part, la persistance de la panniculite après l'augmentation des doses de corticoïde et sa disparition à l'arrêt du MTX sont des arguments chronologiques et évolutifs qui plaident en faveur de l'origine iatrogène médicamenteuse.

## Conclusion

Notre observation est particulière par la survenue d'une panniculite chez une patiente ayant une DM traitée par du MTX et illustre la difficulté diagnostique puisqu'il n'existe aucun signe pathognomonique tranchant en faveur de la DM ou de l'origine iatrogène. L'imputabilité du MTX se basait sur des critères chronologiques et l’évolution ultérieure à l'arrêt du médicament. Cette éventualité doit être connue malgré son caractère exceptionnel puisque l'arrêt du MTX induit en général la disparition des nodules sous cutanés.
