# Erratum: Panniculite au cours d’un traitement d’une dermatomyosite par du méthotrexate

**DOI:** 10.11604/pamj.2020.36.57.23475

**Published:** 2020-06-02

**Authors:** The Pan African Medical Journal

**Affiliations:** 1 The Pan African Medical Journal, Yaoundé, Cameroon

**Keywords:** Erratum, dermatomyositis, panniculitis, méthotrexate, Erratum, dermatomyositis, panniculitis, methotrexate

## Abstract

Cet erratum corrige l’article original: “ Panniculite au cours d’un traitement d’une dermatomyosite par du méthotrexate ”. The Pan African Medical Journal. 2016;23:149. doi:10.11604/pamj.2016.23.149.8950.

## Abstract (en)

This erratum modifies the original article: “ Panniculitis in patient undergoing treatment for dermatomyositis with methotrexate ”. The Pan African Medical Journal. 2016;23:149.doi:10.11604/pamj.2016.23.149.8950.

## Corrigendum

Dans la version originale de l’article, l’orthographe du nom de l’auteur correspondant est incorrecte [1]. Cela a été corrigé à la fois dans la version PDF et HTML dudit manuscrit. Le nom de l’auteur correspondant a été modifié en Nabil Belfeki au lieu de Nabil Bel Feki.
